# 

**Published:** 1884-01

**Authors:** 


					﻿Whole Number,
; CCCCLXVIII.
January, 1884.
Number 1,
Vol. XLVIII.
THE
CHICAGO
MEDICAL
Journal & Examiner.
CHICAGO:
PUBLISHED BY THE CHICAGO MEDICAL PRESS ASSOCIATION
188 Clark Street.
®~Read the Notice of this Journal among Advertisements
				

